# Dietary Flavonoids as Modulators of Lipid Metabolism in Poultry

**DOI:** 10.3389/fphys.2022.863860

**Published:** 2022-04-25

**Authors:** Zhendong Tan, Bailey Halter, Dongmin Liu, Elizabeth R. Gilbert, Mark A. Cline

**Affiliations:** ^1^ Department of Animal and Poultry Sciences, Blacksburg, VA, United States; ^2^ Department of Human Nutrition, Foods, and Exercise, Blacksburg, VA, United States

**Keywords:** lipid, poultry, adipose tissue, flavonoid, feed additive, phytochemical, polyphenols, broiler

## Abstract

Flavonoids, naturally-occurring compounds with multiple phenolic structures, are the most widely distributed phytochemicals in the plant kingdom, and are mainly found in vegetables, fruits, grains, roots, herbs, and tea and red wine products. Flavonoids have health-promoting effects and are indispensable compounds in nutritional and pharmaceutical (i.e., nutraceutical) applications. Among the demonstrated bioactive effects of flavonoids are anti-oxidant, anti-inflammatory, and anti-microbial in a range of research models. Through dietary formulation strategies, numerous flavonoids provide the ability to support bird health while improving the nutritional quality of poultry meat and eggs by changing the profile of fatty acids and reducing cholesterol content. A number of such compounds have been shown to inhibit adipogenesis, and promote lipolysis and apoptosis in adipose tissue cells, and thereby have the potential to affect fat accretion in poultry at various ages and stages of production. Antioxidant and anti-inflammatory properties contribute to animal health by preventing free radical damage in tissues and ameliorating inflammation in adipose tissue, which are concerns in broiler breeders and laying hens. In this review, we summarize the progress in understanding the effects of dietary flavonoids on lipid metabolism and fat deposition in poultry, and discuss the associated physiological mechanisms.

## Introduction

Phytochemicals are natural compounds that are obtained from plants. Phytochemicals are classified into the alkaloids, polyphenols, terpenoids, carotenoids, organo-sulfurs, phytosterols, limonoids, glucosinolates, and fibers, which are further divided into many subtypes according to their chemical structures and characteristics (González-Castejón and Rodriguez-Casado, 2011). Flavonoids are functional derivatives of polyphenols which are the most abundant phytochemicals and are considered to have the greatest health benefits. Results from clinical studies have shown that polyphenols are potent at preventing or slowing the onset of chronic diseases, especially those induced by oxidative stress such as cardiovascular diseases (CVD) and metabolic disorders (Manach et al., 2005).

Flavonoids are a category of plant-derived low-molecular weight secondary metabolites with diverse structures and chemistries. Different from the function of primary plant metabolites (e.g., protein, fat, and carbohydrates which mainly regulate energy metabolism and cell physiology), secondary metabolites are associated with non-nutritive dietary fiber, and are important for interactions between plants and the environment, for instance in conferring resistance to parasites, fungi, and other microorganisms (Leitzmann, 2016). For a long time, flavonoids were considered to be natural toxins. Considering that many flavonoids indeed have adverse effects which depend on their concentration and form in the feed, such as decreasing nutrient availability and inhibiting digestive enzyme activities, they were long characterized as “anti-nutritive” metabolites by nutritionists (Salunkhe et al., 1982). However, through in-depth studies involving high-precision analytical methods and a comprehensive array of cell and tissue culture and animal models, the beneficial bioactive effects of flavonoids have been increasingly recognized and exploited for nutritional and pharmaceutical purposes (Dillard and German, 2000).

Flavonoids constitute a major family of phytochemicals and are generally used as nutraceuticals in foods and food supplements, or exist in a low-concentration form in herbs, teas, beans, vegetables, and fruits where they still have the potential to exert a physiological effect. The majority of research on flavonoids is conducted to explore antioxidant and anti-inflammatory activities. Although flavonoids are considered to be anti-nutritive agents, there is a strong relationship between these phytochemicals and chronic diseases, especially obesity, CVD, and cancer (Craig, 1997; Dillard and German, 2000; González-Castejón and Rodriguez-Casado, 2011). Consumption of vegetables and fruits is linked to a lower risk of obesity and cancer, and some of the underlying mechanisms may involve bioactive flavonoids (He et al., 2004; McMillan et al., 2006; Key, 2011). For instance, there has been much research on the use of dietary phytochemicals as an anti-obesity strategy, as many of them are known to prevent the expansion of adipose tissue through inhibition of the differentiation of preadipocytes into adipocytes, and promotion of lipolysis and apoptosis in mature adipocytes. Among them, flavonoids targeting the adipocyte life cycle decrease preadipocyte proliferation (Rayalam et al., 2008). In addition, the phenolic constituents of flavonoids are known to inhibit the growth of adipose tissue via anti-angiogenesis and metabolic-regulating pathways in various cells (Ejaz et al., 2009; González-Castejón and Rodriguez-Casado, 2011; Sakuma et al., 2017).

In recent years, there has been a growing awareness of the purported health benefits of plant-based foods. However, the benefits of dietary supplementation of polyphenols in agriculturally-relevant species, particularly in commercial poultry production, have not been as well studied. In this review we summarize the known effects of flavonoids, a sub-class of polyphenols, on lipid metabolism and deposition in poultry, and discuss the proposed physiological mechanisms and practical applications.

## Lipid Metabolism and Adipose Tissue Physiology in Poultry

The poultry industry is mainly focused on producing eggs and meat. These products are closely related to lipid metabolism and deposition. In nearly half a century, poultry producers, especially of broiler chickens, have shortened the amount of time required to achieve the desired final body weight (BW), and increased the final body weight for slaughter from 1.5 kg in 70 days to 2.5 kg in 42 days (Havenstein et al., 2003). Modern poultry production drastically increased the body weight gain (BWG), carcass yield, breast weight, and egg production of poultry to satisfy the increasing consumer demand (Wang et al., 2012; Fouad and El-Senousey, 2014), but fat deposition in the abdomen was initially ignored in some breeding schemes, which resulted in high rates of lipid biosynthesis and accumulation as adipose tissue. This seems to be universal that selection of BWG leads to an increase in fat deposition. The modern strains of broilers, for example, contain 15–20% of their BW as fat, more than 85% of which is not required for physiological function (Choct et al., 2000). Nowadays, reducing fat content can be achieved by changing the selection strategy in feed conversion ratio (FCR). Selection for BWG leads to a higher fat content of the carcass, whereas lower FCR tends to produce carcasses with lower fat and higher water content (Pym and Solvyns, 1979; Tůmová and Teimouri, 2010). Although reducing fat content during the selection and breeding process can improve reproductive traits of broilers (Zhang et al., 2018), the disadvantages are also obvious. Since fat is a high heritability trait, excessive pursuit of reduced fat during production has adverse effects on live performance of chickens, especially during the chick-rearing stage (Tůmová and Teimouri, 2010). Thus, excess fat deposits, consequently, remain a major problem in modern poultry production. For producers, excess fat content is sometimes unacceptable, as it is considered to be a waste of dietary energy, while reducing carcass yield and affecting meat quality (which may or may not be acceptable to consumers) (Rabie and Szilágyi, 1998; Fouad and El-Senousey, 2014). As for laying hens and broiler breeders, excess fat deposition significantly impacts metabolic health and reproductive performance (Xing et al., 2009). It is important to note that excess energy acquisition is directly related to food intake, which also continues to be a concern in poultry production. Broiler breeders, in particular, are feed-restricted to achieve target BW’s and fat percentages in order to slow growth and prevent the onset of metabolic disorders which would adversely affect bird health and reproductive output.

Research on lipid metabolism and adipose tissue physiology in poultry has mainly focused on the conversion of feed energy to fat deposition in meat and eggs, and at present, lipid metabolism and related mechanisms are still popular areas of research because fat deposition in poultry products directly affects production efficiency and profitability. The fat content of poultry products, as well as the fatty acid composition, are particularly important to the health of consumers and poultry (Hargis and Van Elswyk, 1993). Because high fat, saturated fatty acids (SFA), and cholesterol are linked to chronic diseases and cancer (Cross et al., 2010; Ferguson, 2010; Santarelli et al., 2010), there is increasing focus on health outcomes associated with consuming poultry meat and products.

For avian species, the main factors affecting lipid deposition include dietary energy, protein, amino acids, and mineral levels. Oils and fats are the most concentrated sources of energy, which lower heat increment, increase the absorption of fat-soluble vitamins, enhance gastrointestinal passage, and can improve palatability (Krogdahl, 1985). The amount of body fat that poultry species accumulate depends on the availability of plasma lipid substrates available from feed and those synthesized in the liver via *de novo* lipogenesis (D'Mello, 2000). Moreover, for laying hens, dietary reductions in fat may lower the cholesterol content of egg yolk and thereby lower the risk of cardiovascular diseases in consumers (Han et al., 1993). Thus, dietary lipid sources and quantity can directly affect fat deposition and composition in poultry. In addition, hormones like insulin regulate lipid metabolism in poultry liver and adipose tissue (Goodridge, 1973; Gross and Mialhe, 1984; Wilson et al., 1986), which coordinately determine the amount of fat that is stored and oxidized in the body.

The liver is the one of the most important organs for lipid metabolism in the body, and is responsible for fatty acid synthesis (in birds and humans), bile synthesis, packaging of lipids for transport, lipid storage and oxidation, and also ketogenesis from lipid substrates when glucose is scarce (Bickerstaffe et al., 1970; Griminger, 1986; Nguyen et al., 2008). Hepatic fatty acid metabolism has been recognized as a major determinant of the changes in blood triglyceride levels and fat deposition in poultry (Guillou et al., 2008).

Adipose tissue, on the other hand, is primarily used for fat storage in birds, and plasma non-esterified fatty acids generally represent liberation of fatty acids from adipose tissue triacylglycerol (TG) stores when the animal is in a fasting state (Griffin et al., 1992). In chicken embryos, the subcutaneous depot is the first to arise during development, and at hatch subcutaneous and neck fat (including above the breast) are relatively well-developed and available as a source of energy and insulation, whereas abdominal fat is largely absent. The abdominal fat pad has a faster growth rate compared to other fatty tissues and ultimately becomes a direct determinant of body fat content (Butterwith, 1989).

In most mammals, there are two types of adipose tissue according to their physical function and color, white and brown, with brown associated with non-shivering thermogenesis due to the action of uncoupling protein-1 (UCP-1) (Oelkrug et al., 2015). Due to the lack of orthologous UCP-1 in the avian genomes, the presence of an equivalent brown fat in birds has been questioned and generally thought to not exist (Saarela et al., 1991). White adipocytes are characterized by the presence of a single, large lipid droplet containing TGs that are liberated via the sequential activation of lipases in the cell (Reue, 2011). Adipocytes originate from mesenchymal stem cells that can be committed to become preadipocytes which are then stimulated to differentiate into the mature adipocyte. There are a multitude of transcription factors and enzymes that are associated with cellular differentiation and the accompanying lipid synthesis, respectively, and these encoded genes tend to be the target of research studies aimed at evaluating how various factors, including dietary supplements, influence adipose tissue development and maintenance (Varga et al., 2011). For brevity, this review will mainly focus on the effects of dietary flavonoids on lipid metabolism and adipose tissue physiology in chickens, with an emphasis on the physiological mechanisms. It should also be recognized that these compounds may also exert direct or indirect effects on appetite regulation, which would in turn influence body composition. Where relevant, such mechanisms will be discussed.

## Flavonoids

Flavonoids are a group of polyphenols that have been widely used in the pharmaceutical and cosmetic industries because of their antioxidant, anti-inflammatory, anti-microbial, and anticarcinogenic effects, (Panche et al., 2016). In the plant kingdom, flavonoids are essential for plant growth, pigmentation, and resistance to plaques, and represent one of the most common groups of compounds in higher plants (Havsteen, 2002). In addition to plants, flavonoids are commonly found in plant-derived foods and beverages, such as fruits, vegetables, tea, cocoa, and red wine (Panche et al., 2016).

**FIGURE 1:**
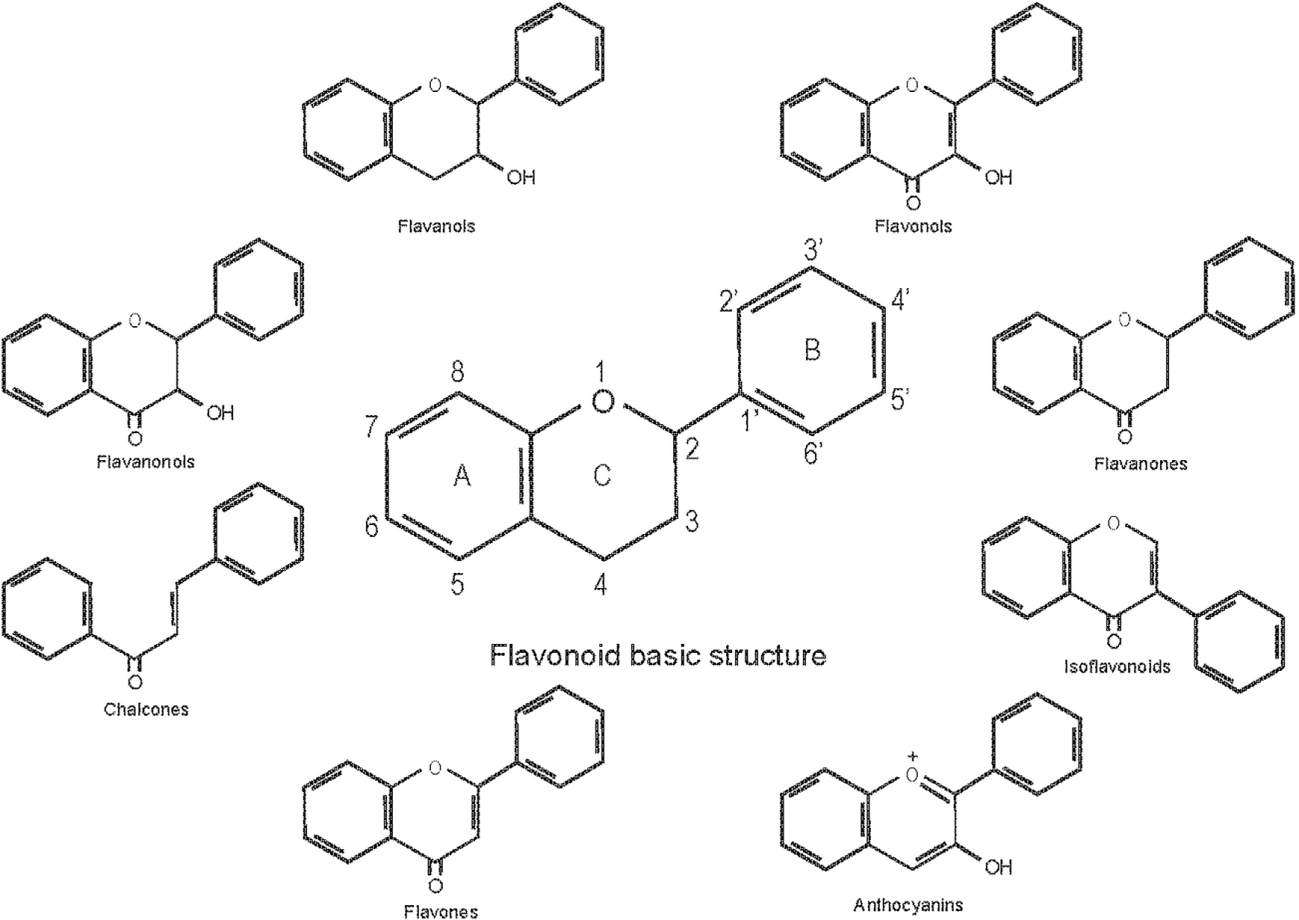
Classification of flavonoids and their basic structure.

Flavonoids are a general term for a series of compounds with a c6-c3-c6 structure (A, B, C ring, Figure 1), which consists of two benzene rings (A, B) linked via a heterocyclic pyrane C-ring (Pietta, 2000). Flavonoids can be divided into various subgroups according to the degree of heterocyclic pyrane C-ring oxidation and the substitution pattern of a functional group on the C ring with a methyl, hydroxyl, glycan, acetyl or other group, the location of the B ring connection (position 2, 3, or 4 of the C ring), and whether the heterocyclic pyrane C-ring forms a ring. The connections of the B ring to the position 3 of the C ring are called isoflavones, and the connections to the position 4 of the C ring are called neo-flavonoids. Those in which the B ring connects to position 2, can be divided into seven subclasses: flavones, flavonols, flavanones, flavanonols, flavanols or catechins, anthocyanins, and chalcones (Panche et al., 2016).

Challenge conditions like oxidative stress (including that caused by heat stress) and diseases have a series of negative impacts on the production performance and welfare of poultry. In environments outside of the comfort zone, birds experience reductions in food intake and depending on the level of severity, cellular dysfunction and lipid peroxidation, leading to associated pathologies (Puthpongsiriporn et al., 2001; Silva et al., 2002). Biological activities of flavonoids have aroused interest for alleviating the detrimental effects of oxidative stress. For example, Peña et al. evaluated the effects of citric flavonoids (quercetin and rutin) combined with ascorbic acid (AA) on the performance and meat quality characteristics of broilers under heat stress for 32 days. They found that the addition of graded AA and citric flavonoids did not affect meat production and quality. However, FCR was improved during the first week post-hatch with 0 and 250 g/ton AA+ citric flavonoids (Peña et al., 2008). The beneficial mechanism is thought to be the stimulation of the expression of stress response proteins and antioxidant enzymes, thereby offsetting production of reactive oxygen species (ROS). Treatment with some flavonoids, like epigallocatechin gallate (EGCG), upregulated the expression of superoxide dismutase to improve the antioxidant capacity and mitigate the influence of heat stress (Hu et al., 2019).

**FIGURE 2:**
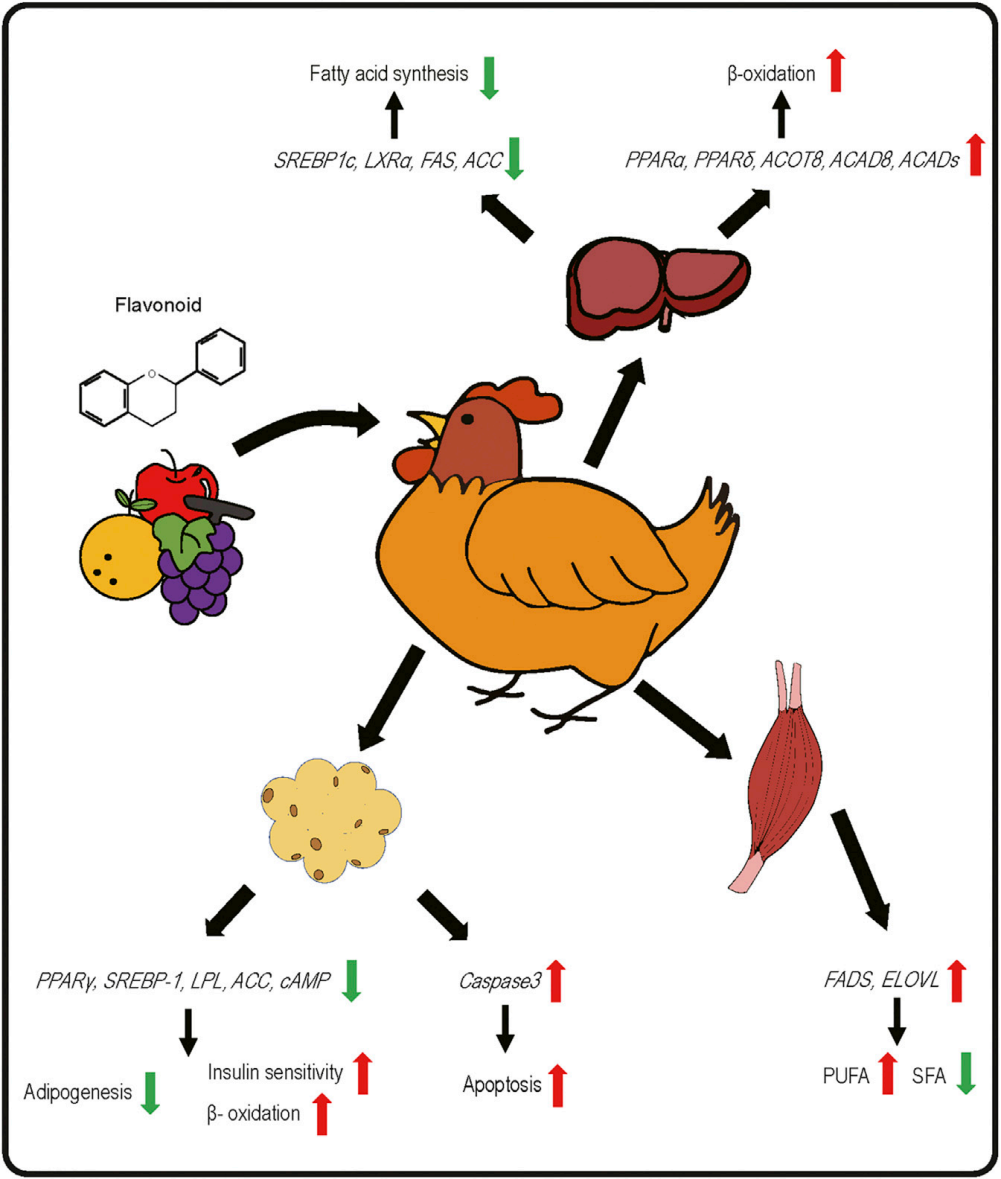
Effects of dietary flavonoids on gene expression related to lipid metabolic pathways in liver, skeletal muscle, and adipose tissue of poultry species.

Two major challenges associated with using dietary flavonoids as a strategy to modulate bird health and growth are 1) that the *in vivo* targets are still unclear for many of these chemicals and results from controlled cell culture studies may not be physiologically relevant and 2) despite much research on their biochemistry, bioavailability continues to be poor. In terms of bioavailability, flavonoids are subject to modification by the intestinal microflora and typical modifications include methylation, glucuronidation, sulfonation and others, as reviewed in (Hu et al., 2017). It is thought that flavonoids exploit nutrient transporters for uptake across the mucosal layer and that following uptake, flavonoids enter the portal circulation and are thus subjected to first-pass metabolism by liver Phase II metabolic enzymes, leading to further modification and potential reductions in cellular bioavailability. Improving bioavailability and absorption are the ultimate goals for utilizing flavonoids as feed supplements. The majority of flavonoids in plants are bound to sugars through a glycosidic bond, known as a glycoside. The glycoside form of flavonoids is not absorbed and must be hydrolyzed in the host intestine. In poultry, flavonoids are mostly absorbed in the ileum at pH 5.0 to 6.8 (Kamboh et al., 2019), although the chemical structure affects their bioavailability, absorption, interaction with cell receptors and enzymes, etc (Abdel‐Moneim et al., 2020). Dietary supplementation of exogenous enzymes (e.g., carbohydrases and tannases) facilitates the breakdown of catechins into smaller monomeric and dimeric units at the cost of reduced digestibility of monomeric and dimeric catechins, suggesting that polymeric structures can improve intestinal utilization of monomeric and dimeric catechins (Chamorro et al., 2017). Among all flavonoids, isoflavones have the highest bioavailability. Anthocyanins are rapidly absorbed, but their bioavailability is the lowest of all flavonoids (Rafiei and Khajali, 2021). Aglycones (non-sugar moiety) tend to be more bioavailable than the corresponding glycoside form (Thilakarathna and Rupasinghe, 2013). For instance, Steensma et al. found that aglycone genistein is more bioavailable compared to glycoside genistein in rats (Steensma et al., 2006). Thus, an important consideration for dietary supplementation of flavonoids is the potential for transformation into chemically distinct molecules with different bioavailabilities and pharmacodynamic properties in the body than the chemicals that were present in the diet. Despite these limitations, there are a plethora of data demonstrating beneficial effects on poultry growth and health, and in particular regulation of lipid metabolism and fat deposition (Table 1). In the remainder of this review, where appropriate, cellular targets of flavonoids will be described and the current state of knowledge discussed, including gaps in our current understanding of flavonoid biology (Figure 2).

**TABLE 1:** Flavonoids and effects on body fat, meat quality/composition and egg quality in poultry.

Species	Chemical class	Results of supplementation	References
Broiler	Flavones	Flavones of sea buckthorn fruits at 0.05, 0.10, and 0.15%, ↓ abdominal fat pad weight, ↑ intramuscular fat in breast	Ma et al. (2015)
		0.1 and 0.2% sea buckthorn flavones ↓ abdominal fat %, drip loss of breast and thigh muscle, and serum triglycerides	Li et al. (2008)
	Flavonols	0.3% or 0.6% kaempferol ↓ abdominal and subcutaneous fat, and plasma and hepatic cholesterol and triglycerides	Xiao et al. (2012)
		0.5 and 1 g/kg quercetin ↑ meat lightness (L*), redness, and oxidative stability during refrigeration for 3 and 9 days	Goliomytis et al. (2014b)
		Quercetin at 200 mg/kg ↓ oleic, palmitic, linoleic, and stearic acid in breast	Oskoueian et al. (2013)
	Isoflavones	5 mg of genistein and 20 mg of hesperidin/kg ↓ muscle fat	Kamboh et al. (2018)
		20 mg/kg of genistein and hesperidin ↑ meat-holding capacity and ↓ lipid oxidation of breast at 0 and 15 days of refrigeration	Kamboh and Zhu, (2013a)
		40 and 80 mg/kg soy isoflavones ↑ L*, water-holding, pH, and catalase and superoxide dismutase activity in breast, and ↓ lipid peroxidation	Jiang et al. (2007)
		Genistein (5 mg/kg) ↓ ratio of 14:0, 18:0, and ΣSFA and cholesterol in breast muscle	Kamboh and Zhu, (2013b)
	Anthocyanins	Anthocyanin fortified barley at 5% ↑ abdominal fat	Park et al. (2012)
		10 and 20% *Tridax procumbens* powder ↑ breast meat juiciness and flavor	Ahossi et al. (2016)
		Konini wheat (∼14 mg/g of anthocyanin) ↑ BWG.	Stastnik et al. (2016)
		Cranberry fruit extract (40, 80, or 160 mg/kg anthocyanin) ↑ feed efficiency and BW.	Leusink et al. (2010)
	Flavanones	Hesperidin and naringenin at 0.75 or 1.5 g/kg ↑ PUFA and n-6, while ↓ SFA in abdominal fat, breast and thigh muscles	Hager-Theodorides et al. (2021)
		0.75 and 1.5 g/kg hesperidin and naringin ↑ breast muscle redness (a*), and ↓ lipid oxidation for 9 days at 4°C and for 120 days at -20°C	Goliomytis et al. (2015)
		Hesperidin and naringenin at 0.75 and 1.5 mg/kg ↑ content of PUFA, omega n-6, and PUFA/SFA ratio in breast muscle and fat	Hager-Theodorides et al. (2021)
		Hesperidin at 20 mg/kg ↓ cholesterol and changed fatty acids (proportions of 18:0, 9c18:1, 20:4n-6 and Σn-3 were ↓) in breast	Kamboh and Zhu, (2013b)
	Flavanols	Green tea powder (0.5, 1 and 1.5%) ↓ % of abdominal fat	Hrnčár and Bujko, (2017)
		Green tea powder at 0.5, 0.75, 1.0 or 1.5% ↓ liver and serum cholesterol and abdominal fat	Biswas and Wakita, (2001b)
	Flavanonols	120 mg/kg taxifolin ↓ lipid peroxidation in fresh meat and during refrigerated storage for 1 month at -18°C	Bakalivanova and Kaloyanov, (2012)
Layer	Flavonols	Quercetin at 0, 0.2, 0.4, or 0.6 g/kg ↑ laying rate and ↓ feed-egg ratio. Haugh unit, eggshell strength and thickness, and yolk protein ↑, while yolk cholesterol ↓	Liu et al. (2013)
	Isoflavones	10, 50, and 100 mg/kg daidzein for 8 weeks ↑ eggshell thickness, percentage, strength, and egg yolk superoxide dismutase	Cai et al. (2013)
	Anthocyanins	8 weeks anthocyanin-fortified barley ↑ laying performance and egg quality	Choe et al. (2013)
		Purple wheat grain at end of laying period in 69 weeks ↑ eggs and yolk weight	Roztočilová et al. (2018)
	Flavanones	0.05% hesperetin, 0.05% naringenin, and 0.5% galacturonic acid ↓ egg yolk cholesterol and ↑ yolk weight and ratio of yolk weight/egg weight	Lien et al. (2008)
		9% dried orange pulp (0.767 g hesperidin and 0.002 g naringin) for a month ↑ yolk oxidative stability and egg shelf life. ↓ performance and egg quality parameters	Goliomytis et al. (2018)
	Flavanols	0.2 and 0.4% green tea catechins ↓ egg weight, specific gravity, thickness and yolk malondialdehyde	Kara et al. (2016)
		Green tea powder (2, 4, 6, 8 g/kg) or tea catechins (0.5, 1, 1.5, 2 g/kg) for 60 days ↓ total cholesterol, triglyceride, and LDL levels in plasma and breast and thigh meat	Zhou et al. (2012)
	Chalcones	5 or 10 g tomato powder/kg ↑ egg production, egg weight and yolk color	Akdemir et al. (2012)
		Pre-injection of 25 mg chalcones ↓ eggs with blood spots	Bigland et al. (1964)
Quail	Flavones	0.1, 1.0 and 10.0 mg morin/scaled quail/day for 8 weeks: no effect on reproduction	Cain et al. (1987)
	Isoflavones	400 or 800 mg of genistein/kg for 90 days ↑ egg production, egg weight, Haugh unit, shell thickness and weight, while ↓ yolk malondialdehyde	Akdemir and Sahin, (2009)
		400 or 800 mg of soy isoflavones/kg ↑ egg quality	Sahin et al. (2007)
	Anthocyanins	Purple field corn ↑ egg quality, performance and carcass quality	Amnueysit et al. (2010)
	Flavanols	0.25, 0.50 and 0.75% powdered green tea flowers ↑ FCR.	Abdel-Azeem, (2005)

### Flavones

Flavones are characterized by a double bond at positions 2 and 3 of the C ring, and a ketone group at position 4 (Figure 1). Most flavones in nature have a hydroxyl group in the A ring at position 5, and depending on the classification of fruits and vegetables, may also have hydroxyl groups in ring A at position 7 and in the B ring at positions 3 and 4. Flavones are abundant in plant branches, leaves and fruits, in the form of glucosides. Celery, parsley, red peppers, ginkgo biloba, and herbs are the main plant sources, and examples of flavones include baicalein, baicalin, diosmetin, luteolin, apigenin, and tangeritin.

Ma et al. (Ma et al., 2015) found that adding different levels (0, 0.05, 0.10, or 0.15%) of sea buckthorn fruits (FSBF), which are rich in flavones, to broiler diets from 1 to 42 days of age, effectively reduced the final abdominal fat pad weight by nearly 20%, while increasing intramuscular fat (IMF) content in breast muscle. Cholesterol, TG, and low-density lipoprotein cholesterol (LDL-C) levels were also reduced. These findings are consistent with those of Li et al. (Li et al., 2008), who also reported that flavone supplementation reduced abdominal fat percentage in chickens. Body weight gain increased and FCR decreased (increased efficiency) from 7 to 42 days when broilers were fed diets supplemented with 100 and 200 mg/kg flavone baicalein (Zhou et al., 2019). Meanwhile, the serum levels of total cholesterol (TC), the ratio of non-high density lipoprotein (HDL)-C/HDL-C, LDL-C/HDL-C, TC/HDL-C, LDL-C, and TGs were decreased after baicalein treatment (Zhou et al., 2019).

Numerous anti-obesity effects have been attributed to flavone bioactivity. Flavone luteolin is a potent TG lipase inhibitor in preadipocytes and enhances insulin sensitivity through activation of peroxisome proliferator-activated receptor γ (PPARγ) (Zheng et al., 2010). Additionally, flavones can suppress obesity-associated inflammation by blunting the nuclear factor kappa-B (NF-κB)-mediated pathway (González-Castejón and Rodriguez-Casado, 2011). Another flavone, baicalein, also functions as an antioxidant and anti-lipase agent. Xiao et al. (Xiao et al., 2021) supplemented the starter diet of Hubbard × Cobb-500 day-of-hatch broiler chicks with 125, 250, or 500 mg/kg baicalein for 6 days, which significantly reduced chick breast muscle and subcutaneous and abdominal fat weights. Expression of mRNAs for factors involved in adipogenesis and fat storage (PPARγ, diacylglycerol acyltransferase; DGAT2) were more highly expressed in the subcutaneous than abdominal fat depot but were not affected by diet. In cell culture studies, mRNA expression of genes related to adipogenesis and lipid accumulation (CCAAT/enhancer-binding protein beta; C/EBPβ, CCAAT/enhancer-binding protein alpha; C/EBPα, sterol regulatory element-binding transcription factor 1; SREBP1, Diacylglycerol acyltransferase; DGAT1, and PPARγ) were generally decreased after baicalein treatment in a dose-dependent manner (3.125, 6.25, and 12.5 μM) or in response to a single dose (50 μM) (Seo et al., 2014; Nakao et al., 2016).

### Flavonols

Flavonols have a C2-C3 double bond, a ketone group in the fourth position of the C ring, and an additional hydroxyl group in the C-ring at position 3. Flavonols, which have the 3-hydroxyflavone backbone, are found in a variety of vegetables and fruits. They are abundant in apples, grapes, lettuce, broccoli, and onions. In addition to vegetables and fruits, other important sources include tea, red wine, and medicinal herbs. Flavonols that have been extensively studied for biological effects in animals are quercetin, kaempferol, myricetin, and fisetin. Because of diverse patterns of methylation and hydroxylation, flavonols are considered to be the most common and largest subgroup of flavonoids (El Gharras, 2009).

Quercetin is reported to exert a variety of biological effects, such as growth promotion, anti-infection, antioxidant, and antiviral, in livestock and poultry species (Comalada et al., 2006; Goliomytis et al., 2014a; Saeed et al., 2017a). It is worth noting that quercetin has anti-obesity effects that are mediated through several pathways that collectively reduce fat accumulation: 1) enhancement of cyclic adenosine monophosphate (cAMP) levels by inactivating phosphodiesterase, to prolong lipolysis in adipocytes; 2) inhibition of insulin receptor and SREBP-1, thereby repressing lipoprotein lipase (LPL), acetyl-CoA carboxylase (ACC), glucose uptake and fatty acid synthesis, and reducing fatty acid absorption and lipid accumulation in tissue; 3) at the transcriptome level, increasing mRNA expression of *Caspase3* and decreasing *PPARγ*, resulting in the apoptosis of adipocytes and inhibition of adipogenesis, respectively, collectively resulting in a reduction in the number of adipocytes (Saeed et al., 2017a). In primary human adipocytes and 3T3-L1 murine adipocytes, quercetin alone or in combination with genistein (isoflavone) and resveratrol (stilbene) reduced the activity of glucose 3-phosphate dehydrogenase (contributor of the glycerol backbone to TG synthesis), thereby inhibiting the late-stage differentiation of adipocytes and promoting the apoptosis of mature adipocytes (Park et al., 2008).

In poultry, dietary supplementation of quercetin was associated with changes in broiler growth and meat composition. Sohaib et al. (Sohaib et al., 2015) showed that adding quercetin to broiler feed reduced the concentration of fatty acids and improved the quality and consumer acceptance of meat products. In addition, quercetin in combination with α-tocopherol significantly increased BWG and reduced SFA while monounsaturated fatty acid (MUFA) content was not significantly altered (Sohaib et al., 2015). Consistent with these findings, Oskoueian et al. (Oskoueian et al., 2013) reported that dietary quercetin supplementation at 200 mg/kg reduced oleic (18:1n-9), palmitic (16:0, 26.1–27.9%), linoleic (18:2n-6) and stearic acid (18:0) in broiler pectoralis major muscle. While it is unclear how quercetin modulates fatty acid composition in the meat, the results from these studies suggest that quercetin has the potential to be used as a dietary additive for broilers to improve growth performance and meat quality.

Quercetin is also an effective functional dietary additive for laying hens. At a concentration of 0.4 g/kg of the basal diet, quercetin not only improved the laying rate, reduced the feed-egg ratio, but also increased the shell strength, shell thickness, and egg yolk protein content throughout the 8 weeks experimental period (Liu et al., 2013). Notably, quercetin has been shown to lower cholesterol and TG levels in the yolk. Kim et al. (Kim et al., 2003) believed that the reduction of cholesterol in egg yolk by quercetin treatment might be due to the inhibition of the activity of HMG-CoA reductase, the rate-limiting enzyme in endogenous cholesterol synthesis. It was also suggested that quercetin modulates intestinal function, altering the composition of the gut microbiome and the luminal microenvironment proximal to the absorption of nutrients (Saeed et al., 2017a). Liu et al. (Liu et al., 2014) studied the effects of quercetin supplementation on the cecal microflora of laying hens, and observed that quercetin (0.2, 0.4, and 0.6 g/kg of diet for 8 weeks) reduced the total number of aerobe and coliform bacteria, whereas the number of beneficial *bifidobacterium* was increased. The results showed that quercetin may function as a metabolic prebiotic and exert important influences on the intestinal environment by regulating the composition of the cecal microflora. Although beyond the scope of this review, it is important to acknowledge the contribution of the gut-microbiome-brain axis to physiology and that dietary changes that influence the microbiome have the potential to in turn influence physiology because of the contribution of bacterial metabolites to host cellular function. Indeed, in both avian and mammalian models, important links have been established between obesity, metabolic syndrome, and the structure and composition of the gut microflora.

Other flavonol compounds also influence fat deposition in poultry. For example, supplementation of kaempferol, which is derived from the rhizome of *Kaempferol galanga L*, inhibited the expression of angiogenesis gene angiopoietin-like 3 (ANGPTL3) in the liver tissue of broilers, while the content of LPL in adipose tissue was elevated (Xiao et al., 2012). As ANGPTL3 inhibits LPL in peripheral tissues and has hypolipidemic effects (Kersten, 2017), it is conceivable that kaempferol could modulate lipid profiles and reduce obesity in broilers. Myricetin, which has insulin-like functions in the body, affects lipid-protein interactions and membrane fluidity, promotes glucose absorption, and stimulates fat generation, and could thus be of therapeutic value in the management of diabetes (Ong and Khoo, 1996). In the study of poultry, myricetin not only accelerated growth, but also acted as a potent antioxidant to protect lipids in the body and post-mortem tissue from oxidation (King et al., 2014).

### Flavanones

Flavanones, also known as dihydroflavones, are ubiquitous in almost all citrus fruits such as oranges, lemons, and pomelos, and are the main source of the bitter taste in the juice. The difference from flavones is that the C ring of flavanones is saturated (Figure 1). Citrus flavanones, like other flavonoids, also possess antioxidant and lipid-lowering properties, and examples in this subclass include naringin, naringenin, hesperidin, and hesperetin.

Addition of hesperidin to the poultry diet can improve antioxidant capacity, health and egg production, reduce serum and yolk cholesterol content in laying hens (S.Ting et al., 2011), and improve the fatty acid profile by reducing SFA and increasing polyunsaturated fatty acids (PUFA) content in broilers (Hager-Theodorides et al., 2021), and bolster the immune response against lipopolysaccharide (LPS) (Kawaguchi et al., 2004). Fotakis et al. (Fotakis et al., 2017) demonstrated that when supplemented in the broiler diet (0.70–1.5 g/kg), hesperidin reduced serum lipid content and significantly increased plasma alanine. Hager-Theodorides et al. (Hager-Theodorides et al., 2021) also found that the addition of hesperidin and naringenin to the broiler diet increased omega n-6 FA and PUFA/SFA ratios in breast muscle and fat pads. An explanation for these observed effects on fatty acid composition is that hesperidin and naringenin can enhance the expression of genes encoding factors related to fatty acid β-oxidation as well as fatty acid synthesis.

In a study with laying hens, Lien et al. (Lien et al., 2008) extracted crude hesperidin (31.5%), crude naringenin (39%), and crude pectin (60%) from citrus and grapefruit peels for supplementation into the diet at concentrations of 0.05, 0.05, and 0.5%, respectively. The results showed that the extracts did not significantly change some traits like egg production, eggshell strength, and eggshell thickness. The concentrations of egg yolk cholesterol, and serum cholesterol and TGs, however, were reduced. Excreta cholesterol levels in the hesperidin (14.18 mg/100 g) and naringenin (16.73 mg/100 g) groups were about double that of the control group (7.43 mg/100 g).

At the transcriptional level, flavanones enhance adipogenesis. Hager-Theodorides et al. (Hager-Theodorides et al., 2021) supplemented 240-day-old Ross 308 broiler chickens with hesperidin (0.75 or 1.5 g/kg feed) and naringenin (0.75 or 1.5 g/kg feed) for a month. They analyzed the fatty acid profile of the abdominal fat pad, and breast and thigh muscles, and found that both hesperidin and naringenin significantly reduced SFA and increased PUFA and n-6 content. They suggested that these effects might be attributed to increased expression of fatty acid β-oxidation related genes (*PPARα* and Acyl-CoA Oxidase 1; *ACOX1*) and the fatty acid synthase (*FASN*) gene. Interestingly, there are two forms of citrus flavanones, the glycosides form (naringin, narirutin, hesperidin) and aglycone form (naringenin, hesperetin). Both forms can be bioconverted through cytolase treatment and have different effects on adipogenesis. Lim et al. (Lim et al., 2015) found that cytolase-treated citrus flavanone, which increased the aglycone form while decreasing the glycosides form, markedly inhibited the differentiation of 3T3-L1 preadipocytes, and naringenin and hesperetin suppressed the protein and mRNA expression of CEBPα, PPARγ, as well as the mRNA levels of SREBP1c. This is inconsistent with the results of Saito et al. (Saito et al., 2009) that flavanone (CAS No. 487-26-3) promoted the differentiation of 3T3-L1 preadipocytes via acting as a PPARγ ligand. Yoshida et al. (Yoshida et al., 2010) showed that naringenin and hesperetin inhibit the ERK and NFκB pathways and reduced free fatty acid (FFA) release and prevented FFA-induced insulin resistance in mouse adipocytes. Thus, multiple studies demonstrate effects of flavonones on lipid metabolism in adipocytes and varying results could be explained by multiple factors, including form and purity of the chemical used, time of treatment application and treatment duration, endpoints for molecular analyses, and passage number and other characteristics of the cell line used.

### Flavanonols

Flavanonols are 3-hydroxyl derivatives of flavanones and are also referred to as dihydroflavones because the double bond between the C ring positions 2 and 3 is hydrogenated. Red onion, vinegars, and red wine contain large amounts of flavanonols.

In cell, *in vivo* animal, and human volunteer health studies, taxifolin shows a wide range of health-promoting effects and biological activities including anti-inflammatory, anti-cardiovascular and anticancer, etc. In a cell culture study using HepG2 (hepatocyte) cells, taxifolin supplementation (200 μM) inhibited cholesterol synthesis, possibly by suppressing HMG-CoA reductase activity (Theriault et al., 2000). A more in-depth study in HepG2 cells found that taxifolin treatment limited the extracellular availability of TGs by inhibiting diacylglycerol acyltransferase (DGAT) and microscopic TG transfer protein (MTP) activities, resulting in a decrease in apolipoprotein B secretion, which is positively correlated with the development of cardiovascular artery disease (Casaschi et al., 2004; Sunil and Xu, 2019). Zhao et al. (Zhao et al., 2018) used streptozotocin-induced diabetic rats as a model and found that taxifolin treatment was associated with a reduction in adipocyte size in the adipose tissue. Studies in poultry have shown that taxifolin (dihydroquercetin) has protective effects on the heart, kidneys, and liver (Saeed et al., 2017b; Cai et al., 2019; Zhang et al., 2019). However, studies regarding the effects of taxifolin on lipid metabolism and adipose tissue physiology in poultry are scarce, and taxifolin appears to function as an antioxidant. Pirgozliev et al. (Pirgozliev et al., 2018) found that feeding taxifolin did not improve the production performance of broilers, except for increasing the redness index of breast meat. Balev et al. (Balev et al., 2015) also found no significant changes in the growth performance of hybrid “Ross” broilers whose diets were supplemented with taxifolin (40 mg/kg body weight) for 49 days.

### Flavanols

Flavanols can also be referred to as flavan-3-ols, which contain a hydroxyl group on the C-ring, however there is no double bond and ketone group at position 4 of the C ring. Flavanols can exist as monomers (catechins) or polymers (pro-anthocyanidins). Flavanols are common in many fruits such as bananas, apples, pears, and a variety of drinks and food such as green tea, red wine, and chocolate. Application of dietary flavanols in poultry production can effectively reduce lipid and cholesterol content in blood and yolk as well as improve production performance, carcass, and meat quality (Aziz and Karboune, 2018; Ogbuewu et al., 2019).

Catechins, which are abundant in green tea, and which include epicatechin (EC), epicatechin gallate (ECG), and EGCG, are well studied for their anti-inflammatory, antioxidant, and anti-obesity effects. Studies in mammalian cell culture and animal obesity models have shown that catechins inhibited adipocyte proliferation and differentiation, lipogenesis, and fat deposition (Wolfram et al., 2006; Abd El-Hack et al., 2020). Kim et al. (Kim et al., 2010) demonstrated that EGCG reduced glycerol-3-phosphate dehydrogenase activity, which hindered TG production because of the lack of a glycerol backbone for esterification. Additionally, EGCG treatment inactivated forkhead transcription factor class O1 (FoxO1) and SREBP1c, which are transcription factors involved in adipocyte differentiation and lipid synthesis. Friedrich et al. (Friedrich et al., 2012) demonstrated that short-term supplementation (up to 1% for 4–7 days) of dietary EGCG to mice inhibited SREBP1c and downregulated its downstream genes *FAS* and stearoyl-coa desaturase (*SCD*) in liver. In cell culture, EGCG at 5 μM inhibited the expression of *PPARγ2* and *C/EBPα* during 3T3-L1 adipocyte differentiation (Furuyashiki et al., 2004). Hung et al. (Hung et al., 2005) found that EGCG (20∼50 μM) also inactivated extracellular signal regulated kinase 1 (*ERK1*) and *ERK2* and arrested 3T3-L1 preadipocytes at the G0/G1 phase. Moreover, EGCG promoted β-oxidation and thermogenesis. Dulloo et al. (Dulloo et al., 2000) demonstrated the synergistic effect of green tea extract (GTE; containing 200 μM EGCG) and caffeine on increasing interscapular BAT thermogenesis. The authors speculated that catechins and caffeine may play important roles in the sympathetically released noradrenaline (NA)-cAMP axis, which may also be responsible for the pronounced effect of GTE on BAT thermogenesis. EGCG directly inhibited catechol-O-methyl-transferase, which catalyzes the degradation of NA, thereby prolonging the action of sympathetically released NA. Caffeine, however, inhibited phosphodiesterase, an enzyme that breaks down cAMP. Thus, EGCG and caffeine synergistically increased the intracellular cAMP levels, which then increased activated PKA-dependent phosphorylation of hormone-sensitive lipase and subsequently free fatty acid release for oxidation. However, this might not be as relevant to the discussion of poultry, because it is questioned whether birds have cells that are analogous to brown adipocytes, due to the absence of uncoupling-protein 1 (UCP-1) in the genome.

It is generally accepted that dietary supplementation of catechins affects gastrointestinal tract absorption of water, glucose, fats, minerals, and amino acids (Koo and Noh, 2007; Frejnagel and Wroblewska, 2010). Plant catechins inhibit the activity of digestive enzymes such as α-amylase, trypsin, chymotrypsin and lipase (Salunkhe, 1982) and also interact with proteins to render them insoluble (Kumar and Singh, 1984). *In vitro* studies have shown that green tea catechins interfere with lipid emulsification, digestion and micelle solubilization, which are key steps involved in the intestinal absorption of dietary fatty acids, cholesterol and other lipids. Green tea or its catechins may also reduce the absorption and tissue accumulation of other lipophilic organic compounds (Koo and Noh, 2007). A number of studies have shown that supplementation of catechins from GTE did not affect broiler FCR or BWG. Kaneko et al. (Kaneko et al., 2001) found that 10 weeks of treatment with dietary inclusion levels of GTE at 1, 2.5 and 5% significantly reduced broiler BWG. Yang et al. (Yang et al., 2003) concluded that antibiotic-free green tea (0.5, 1 and 2%) intake for 6 weeks in Ross broilers did not improve feed intake and feed efficiency, but increased BWG. Shomali et al. (Shomali et al., 2012) noted that 1, 2 and 4% of green tea powder (GTP) in the diet for 2 weeks produced no difference in FCR. However, Erener et al. (Erener et al., 2011) showed that 6 weeks of 0.1 or 0.2 g/kg feed GTE improved feed efficiency and increased BW of 42-day-old broilers. Abdel-Azeem (Abdel-Azeem, 2005) also observed that the addition of 0.25, 0.50 and 0.75% powdered green tea flowers in Japanese quail feed increased FCR. The inconsistent results, which apparently are in contradiction with the well-established effects of GTE’s on nutrient digestion and absorption, may be related to the different catechin components in green tea and the relative abundance of each in various extracts. It is worth noting that differences in broiler strains, inclusion levels, and treatment times between different studies can also lead to significant differences in final results.

In relation to layers, the results of catechin research have also yielded conflicting results. Ariana et al. (Ariana et al., 2011) observed that 0.5% GTE or 1.5% GTP improved egg yield during the later stage of egg production (64–75 weeks of age). Yamane et al. (Yamane et al., 1999) found that 0.67% GTE reduced egg weight, where Biswas and Wakita (Biswas and Wakita, 2001a) observed similar results when utilizing 0.3% GTP. Others have reported that 0.2% green tea leaves improved egg production and egg mass of laying hens aged 20–44 weeks (Al-Harthi, 2004). Again, this could be due to differences in active ingredients and concentrations in green tea, as well as differences in experimental design such as dietary inclusion level, duration of the study, genetic background of the bird, etc. A challenge in implementing GTP additives in poultry is establishing the optimal level of inclusion to promote the desired effects on animal performance. In addition, when using plants (or derivatives), physiological effects can be attributed to multiple chemicals, whose interactions, metabolism, and effects on cells are complex, making it difficult to define the specific “cocktail” of supplements that will yield optimum results.

Notably, addition of green tea catechins to poultry feed can significantly reduce fat accumulation. Hrnčár (Hrnčár and Bujko, 2017) found that 6 weeks of dietary GTP supplementation at 0.5, 1 and 1.5% decreased abdominal fat pad weights in broilers. Biswas and Wakita (Biswas and Wakita, 2001b) studied the growth performance of broilers that were fed *ad libitum* with starter and finisher diets containing 0.5, 0.75, 1.0 or 1.5% GTP. Feed intake was decreased by about 5.1–15.9% in the 1.0% GTP feeding group, while feed conversion in the 5.0–1.0% GTP feeding groups was improved by 4.1–11.4%. The levels of liver cholesterol, liver fat, and serum cholesterol were decreased in GTP treatment groups. The most significant change observed was a 1.65% loss of relative weight of abdominal fat in a dose-dependent manner. Raederstorff et al. (Raederstorff et al., 2003) concluded that green tea’s ability to lower cholesterol was mainly due to EGCG, which could interfere with the micelle dissolution of cholesterol in the digestive tract to regulate lipid digestion, absorption and availability to the liver for bile and lipoprotein synthesis.

Beyond the small intestine, catechin treatment was associated with reduced cholesterol synthesis in the liver (Yousaf et al., 2014). Broilers treated once daily via oral administration of GTP at 50 or 100 mg/kg of BW for a total of 20 days had reduced body fat (Huang et al., 2013). This is partially due to reduced fatty acid synthesis and enhanced β-oxidation. When EGCG was dissolved in water and orally administered to broilers at 80 mg/kg BW for 4 weeks, there was a reduction in serum TGs and LDL, but an increase in HDL cholesterol (Huang et al., 2015). Supplementation of laying hens diet with 1 g/kg green tea catechins for 60 days reduced plasma total cholesterol, TGs, LDL, and body fat content (Zhou et al., 2012). Collectively, data demonstrate that catechins can reduce lipid accumulation in poultry and potentially improve poultry health and meat quality.

Proanthocyanidins, the most structurally complex and abundant dietary flavonoids that are oligomers of epicatechins or catechins, are found in many plants such as apples, grape seeds and skins, cocoa beans, cranberries, and more (de la Iglesia et al., 2010). Such compounds account for the astringent and bitter taste characteristics of fruit (Lesschaeve and Noble, 2005). Although there are large amounts of proanthocyanidins in the diet, it is important to note that they neutralize proteins in the gut to form tannin-protein complexes that reduce nutrient digestion and absorption (Hagerman and Butler, 1981). In terms of lipid metabolism and adipocyte function, grape seed proanthocyanidins can activate the cAMP signaling pathway in 3T3-L1 adipocytes, thereby inducing lipolysis (Pinent et al., 2005). El-Damrawy (El-Damrawy, 2014) observed that 100 and 200 mg/kg grape seed extract (GSE) supplementation for 3 weeks mitigated some of the negative effects of heat stress in broilers. For example, liver superoxide dismutase and glutathione concentrations were increased and heterophil/lymphocyte ratio, plasma corticosterone, TGs, LDL, HDL and liver malondialdehyde were decreased in broilers fed the GSE-supplemented feed. Farahat et al. (Farahat et al., 2017) reported on the effects of grape seed extract (125, 250, 500, 1,000, and 2000 ppm in diet) in broilers from hatch to 42 days. Although there were no differences in growth performance, total lipids, and high and very low-density lipoprotein cholesterol when compared with control groups, the TC and LDL-C levels were decreased after feeding diets that were supplemented with GSE. Roy and Schneeman (Roy and Schneeman, 1981) suggested that proanthocyanidins can bind and inhibit intestinal absorption of cholesterol, thereby reducing cholesterol levels in the body.

### Anthocyanins

Anthocyanins, the glycoside forms of anthocyanidins, are the principal constituent of phyto-pigments of fruits, vegetables, and flowers. The different colors of anthocyanins are usually determined by pH and the methylation or acylation of hydroxyl groups in the A and B rings. They mainly exist in outer cell layers of various fruits such as grapes, strawberries and blueberries, etc. The C ring of anthocyanins has two double bonds, and anthocyanins usually exist in different chemical forms (Figure 1). Cyanidin, delphinidin, peonidin, pelargonidin, and petunidin are common anthocyanidins.

Anthocyanins have various bioactive effects such as inhibiting lipid peroxidation, anti-inflammation, improving cell viability, boosting immunity responses and DNA damage repair (Tsuda et al., 2006; Sivamaruthi et al., 2020). Thus, dietary supplementation of these compounds in animals could potentially achieve many physiological benefits, including protective effects against pathogens and mitigating deleterious effects of chronic heat stress. Csernus et al. (Csernus et al., 2020) reported that 0.5% dietary addition of anthocyanins for 26 days improved the BW, average daily gain and average daily feed intake. In chickens that were fed the supplemented diet and challenged with LPS, there was decreased mRNA expression of splenic and ileal interleukin-1β, increased villus height: crypt depth ratios, and thickened mucosa, indicative of increased absorptive surface area. Stantnik et al. (Stastnik et al., 2014a; Stastnik et al., 2014b) found that Konini wheat containing about 14.01 mg/g anthocyanins and milk thistle seed cakes containing 129.83 mg/kg cyanidin-3-glucoside promoted BWG and improvements in meat quality traits of broilers, respectively. Leusink et al. (Goliomytis et al., 2015) noted that 40, 80, or 160 mg/kg anthocyanins from cranberry fruit extract, supplemented for 5 weeks, had no effects on broiler bird performance, meat properties, general health or intestinal integrity. However, treatment lowered the mortality rate, the populations of *Enterococcus spp*. in cecal and cloacal samples, and had positive effects on feed efficiency and BW. Aditya et al. (Aditya et al., 2018) examined the growth performance of broiler chickens treated with grape pomace (*Vitis vinifera*) (containing 1,134 mg/kg anthocyanin). At different concentrations, grape pomace did not affect BWG, feed intake, or FCR. In addition, serum glucose, TGs, and HDL-C were not affected, but total cholesterol content was significantly decreased and some meat quality parameters were improved.

Although there are no detailed experimental data for poultry adipose tissue, anthocyanins show strong anti-lipid production properties in other experimental models. In obesity-related human studies, consuming red (red-flesh) orange juice that contained large amounts of cyanidins reduced risk factors associated with being overweight or obese, for instance insulin resistance, systolic and diastolic blood pressure that were reduced in volunteers after intervention (Azzini et al., 2017; Sivamaruthi et al., 2020). Silveira et al. (Silveira et al., 2015) found that human subjects who consumed 750 ml of red orange juice per day for 8 weeks showed decreases in serum levels of TC and LDL-C. In the diet of obese individuals, 50 g of carbohydrates were substituted with blueberries which contain large amounts of delphinidin, malvidin, and petunidin. After 12 weeks of nutritional therapy, the blueberry-supplemented group lost weight and had less body fat (Istek and Gurbuz, 2017). In addition, anthocyanins inhibited pro-inflammatory markers in obese subjects (Hogan et al., 2010; Santamarina et al., 2020). The reduction in inflammation is particularly appealing, as persistent low-grade chronic inflammation associated with obesity can cause a number of chronic metabolic diseases. Anthocyanin treatment inhibited the expression of *PPARγ* and *FAS* in high-fat diet-induced obese mice (Wu et al., 2016). *In vitro*, accumulation of TGs in 3T3-L1 preadipocytes was reduced after grape anthocyanin treatment in a dose-dependent manner, due to changes in lipogenic genes such as *LXRα* (*Liver X receptor α*), *SREBP-1c*, *PPARγ*, *C/EBPα*, *FAS*, *SCD-1* and *ACCα* (Lee et al., 2014).

### Chalcones

Chalcones are characterized by the absence of the C ring in the basic flavonoid skeleton structure and are also classified as open-chain flavonoids (Figure 1). Chalco-naringenin, phlorizin, arbutin, and phloretin are the major examples of chalcones. Chalcones are found in tomatoes, pears, apples, strawberries, as well as in wheat products, red wine, and herbs. Chalcones have important pharmacodynamic significance as they are described as potent anti-inflammatory, antioxidant, anti-proliferative, anti-infective, anticancer, and anti-microbial agents in various animal species, including chickens. For example, over 50 years ago, poultry researchers began to inject chalcones into laying hens in order to eliminate the number and size of blood spots present in eggs (Bigland et al., 1965).

The dihydrochalcone phlorizin is abundant in the leaves of sweet tea and is perhaps most well-known to researchers as a competitive inhibitor of the sodium-glucose transporters (SGLT1 and 2). Phlorizin has thus been studied for managing diabetes mellitus and obesity by inhibiting glucose uptake and resorption (Ehrenkranz et al., 2005; Vaidya and Goyal, 2010), but it can be rapidly hydrolyzed into phloretin in the small intestine of mammals, limiting its potential as a therapeutic agent for diabetes. However, Awad et al. (Awad et al., 2007) reported that phlorizin had the same effect on reducing glucose absorption in the jejunum as deoxynivalenol, a common mycotoxin in feedstuffs. Glucose uptake in laying hens was reduced after treatment with 100 µl/ml phlorizin, consistent with Bierbach (Bierbach et al., 1979), where 1 mM phlorizin also reduced glucose absorption in isolated chicken intestinal epithelial cells.

Chalco-naringenin is mainly found in tomatoes and possesses strong anti-inflammatory properties which can be beneficial for relieving chronic inflammation in obese individual adipose tissue. In obesity-associated adipose tissue, chalco-naringenin inhibited proinflammatory cytokines like monocyte chemoattractant protein 1 (MCP1) and TNF-α in the paracrine loop between adipocytes and macrophages, and decreased overall inflammation as well as insulin resistance (Hirai et al., 2010). There is little known on the precise quantification of chalcone and its subclass in feed additives for poultry, and this review discusses relevant additives rich in chalcone, such as tomato powder. In Japanese quail exposed to heat stress-induced conditions, feed intake, BWG, and FCR all increased when fed a diet supplemented with either 2.5 or 5.0% tomato powder for 3 weeks (Sahin et al., 2008). Similarly, laying hens that were fed a diet containing 5 or 10 g/kg of tomato powder for 90 days, displayed significant increases in feed intake yet a decrease in FCR. Other factors such as egg production, egg weight, yolk color (darker color), and duration of egg production were all increased, while there was also a decrease in egg yolk lipid peroxidation (Akdemir et al., 2012). Omri et al. (Omri et al., 2019) reported that the dietary incorporation of 1% tomato paste, 4.5% linseed, and 1% red pepper in Novogen White laying hen feed for 47 days reduced egg yolk content of palmitic acid and stearic acid. Moreover, SFA and the ratio of omega 6- to 3-PUFAs were decreased, and the total content of PUFAs increased. Thus, dietary chalcones may serve as an attractive strategy to mitigate obesity and associated inflammation and metabolic disorders in broiler breeders and laying hens, while also favorably modulating lipid composition in meat and eggs.

### Isoflavones

Isoflavones are unique isomers of flavones, with both glycoside and aglycone forms. Isoflavones are not widely distributed within the plant kingdom, their main presence being in soybean and leguminous plants. Isoflavones in soybeans include genistein, daidzein and glycitein and their respective glycoside forms, with the concentration ratio being about 1:1:0.2 (Manach et al., 2004). Genistein is structurally similar to estrogen and can bind to estrogen receptors, particularly estrogen receptor-beta. Thus, genistein may have weak estrogenic (or antiestrogenic depending on its concentration) properties and is hence referred to as phytoestrogen.

Isoflavones, as a feed supplement, reduced fat deposition, an effect that was attributed to its estrogen-like properties. Gou et al. (Gou et al., 2020) reported that corn–soybean meal-based diets supplemented with linseed oil (2% or 4%) and 30 mg/kg soybean isoflavones reduced the abdominal fat percentage of broilers aged 29–66 days. Adding soybean isoflavones also increased the content of α-linolenic acid (C18: 3n-3), EPA (C20:5n-3) DHA (C22:6n-3) and total n-3 PUFA in breast muscle, while the addition decreased palmitic acid (C16:0), lignoceric acid (C24:0), SFA and n-6: n-3 ratio in breast muscle and total TG, total cholesterol and malondialdehyde content in plasma. Gene expression analysis in broiler breast muscle revealed that the expression of fatty acid desaturase 1 (FADS1), FADS2, elongase 2 (ELOVL2) and ELOVL5 increased in response to soybean isoflavone supplementation. Similarly, Payne et al. (Payne et al., 2001) observed that low crude protein diets with supplemental crystalline amino acids (CP-AA) and a low concentration of soy isoflavones reduced the weights of abdominal fat pads in 9–52 day-old broilers, which were intermediate between chicks fed the corn–soybean meal diet and low CP-AA diet. Thus, these data suggest that the decreased fatness in broilers may be partially attributed to an increased level of soy isoflavones in these diets.

Effects of isoflavones were also observed on egg-laying and egg lipid composition. Corn–soybean meal-based diet supplementation of genistein at 800 mg/kg feed for 90 days significantly improved the antioxidant indices, feed intake and the egg-laying efficiency of quail (Akdemir and Sahin, 2009). Lv et al. (Lv et al., 2018) demonstrated that a relatively low concentration of genistein (40 mg/kg) in a high-energy and low-choline diet for 64 days significantly increased the hypothalamic mRNA expression of gonadotropin releasing hormone (GnRH) mRNA and raised serum estrogen levels, which were associated with improved egg laying performance. Genistein treatment also reduced liver concentrations of long-chain SFA, MUFA, and the n-6: n-3 PUFA ratio. It was suggested that dietary genistein inhibits the expression of fatty acid synthesis-related genes, SREBP1c, LXRα, FAS, and ACC, meanwhile promoting the expression of β-oxidation related genes PPARα, PPARδ, ACOT8, ACAD8, and ACADs. There were differing effects in chickens that received a diet with greater concentrations of genistein (400 mg/kg), further reinforcing that a challenge in widespread implementation of dietary supplementation is identifying optimum dose ranges.

Flavonoids may manipulate the intestinal microbiota and in turn be transformed into relatively more or less bioactive polyphenols, or have beneficial effects by modulating the structure of the microbiome as probiotics that enrich for beneficial species of bacteria (Abbas et al., 2017). A well-studied example is the conversion of soybean isoflavone daidzein to equol, which has strong biological activity as a phytoestrogen. One attractive strategy for improving antioxidant status and establishment of a healthy microbial community is in ovo supplementation during embryonic development. It is beyond the scope of this review to discuss in detail, but depending on the day and location of injection and the diluent (e.g., oils, alcohols, etc.), the flavonoid may be absorbed and exert biological effects during critical stages of development, with physiological changes that persist after hatching. Ni et al. (Ni et al., 2012) injected 20 or 100 μg equol into the albumen on embryonic day 7, and at 49 days post-hatch, equol-treated chickens had lower serum TGs and TC, but greater HDL-C concentrations than controls. These changes were accompanied by differences at the molecular level in the liver, including elevated carnitine palmitoyl transferase 1 and reduced FAS mRNAs. These results suggest that early life exposure to equol may induce changes in the liver that lead to increased fatty acid catabolism and reduced synthesis, thereby reducing circulating levels of liver-derived lipids. Similarly, Wei et al. (Wei et al., 2011) observed that in ovo injection of equol affected female broiler meat quality, including decreased redness (a*), cooking loss, and 24 and 48 h drip loss. Further research is needed to determine the practicality of in ovo supplementation of flavonoids in the poultry industry and robustness of physiological effects.

### Neoflavonoids

Neoflavonoids are relatively uncommon but are reported to exert a multitude of beneficial health effects. They are distributed in *Coutarea hexandra*, *Calophyllum inophyllum* and *Pityrogrammacalomelanos* var. *aureofiava* (Donnelly and Boland, 2017). Dalbergin is the most common neoflavonoid in the plant kingdom. Neoflavonoids are characterized by a 4-phenylchromen backbone without a hydroxyl group substitution at position 2 of the C ring. These compounds have anti-osteoporosis, anti-androgen, anti-inflammatory, anti-tumor, anti-allergy, anti-oxidation and other biological activities (Liu et al., 2017). There are no reports on the effects of dietary neoflavonoids on livestock, poultry production, or on lipid metabolism and deposition.

## Conclusions and Future Directions

In recent years, dietary flavonoids have received considerable attention in poultry research due to their various beneficial effects on health, growth performance, and meat quality. Given that a vast array of such chemicals exist in nature and many flavonoids are antioxidants with anti-inflammatory and other properties, there are possibilities for developing additives using one or more of these natural and cheap compounds to economically promote production while improving health and meat and egg quality. Dietary supplementation of flavonoids can modulate lipid metabolism and deposition, and in particular alter the fatty acid composition and reduce the cholesterol and TG content of poultry meat and eggs. In adipose tissue, some flavonoids inhibit adipogenesis while promoting lipolysis and apoptosis, which collectively prevent the expansion of adipose tissue. As antioxidants, flavonoids can protect other nutrients from oxidation and contribute to a healthy cellular environment at the gut mucosal layer and in other tissues. The anti-inflammatory properties of flavonoids are also an added benefit because obesity, which is a serious issue in broiler breeders and laying hens, is generally associated with a chronic low-grade inflammation, which eventually leads to a host of comorbidities. A challenge in practically applying results of this research is that many studies yield conflicting results regarding consistent effects of flavonoids on growth and production parameters. It is important to note though, that such studies differ in the form of flavonoid supplemented (extracts vs pure compound vs mixture of compounds), doses in the diet, duration of study, genetic background of bird, and processing of ingredients and diet mixing (as well as remaining composition of diet) which can affect the molecular structure, bioavailability, and bioactivity of the flavonoids and other nutrients in the diet. Additional studies are needed at both the cell culture and whole animal level, especially in poultry adipose tissue, skeletal muscle and the liver, in order to reveal the cellular and molecular mechanisms responsible for the effects of flavonoids on lipid metabolism and fat accretion.
